# Reforming *Spartina alterniflora* policies for sustainable coastal resilience: an evidence-based adaptive strategy

**DOI:** 10.1038/s44185-026-00149-2

**Published:** 2026-08-05

**Authors:** Fu Wang, Hong Wang, Qian Yu, Peng Yang, Shu Gao, Yunzhuang Hu, Xueqiang Lu

**Affiliations:** 1 Tianjin Center (North China Center of Geoscience Innovation), CGS Key Laboratory of Coast Geo-Environment, China Geological Survey (CGS), Tianjin, China; 2 Tianjin Key Laboratory of Coast Geological Processes and Environmental Safety, Tianjin, China; 3 Key Laboratory of Coast and Island Development of the Ministry of Education, School of Geography and Ocean Science, Nanjing University, Nanjing, China; 4 Tianjin Key Laboratory of Environmental Technology for Complex Trans-Media Pollution and Tianjin International Joint Research Center for Environmental Biogeochemical Technology, Tianjin, China; 5 College of Environmental Science and Engineering, Nankai University, Tianjin, China

**Keywords:** Ecology, Ecology, Environmental sciences, Environmental social sciences

## Abstract

The global debate on managing invasive *Spartina alterniflora*—eradication versus coexistence—remains unresolved. China’s nationwide eradication campaign risks ignoring regional ecological variations and potential benefits like sediment stabilization and carbon sequestration. We advocate adaptive, site-specific strategies that balance ecological risks with functional contributions, replacing one-size-fits-all eradication with evidence-based management.

*Spartina alterniflora*, a perennial grass native to North America’s East Coast, is a globally invasive species known for rapid growth and competitive dominance. Intentionally introduced to China in 1979 to stabilize mudflats, reinforce seawalls, and support land reclamation, it was first planted in Fujian province^1^. Now spanning over 82,000 hectares across all coastal provinces of mainland China (Fig. 1), *S. alterniflora* threatens biodiversity by outcompeting native plants and altering critical coastal habitats, including tidal flats vital for migratory shorebirds. Its rapid spread, from Europe to Oceania (Fig. 1) (Supplementary S1), underscores a global challenge. Yet *S. alterniflora* also offers benefits, such as sediment accretion and carbon storage, particularly in China’s northern coasts (north of Hangzhou Bay, Fig. 1)^2–6^. Current eradication efforts, relying heavily on physical removal, disrupt ecosystems and fail to address these dual effects. Science-driven, region-specific policies are urgently needed to balance risks and benefits of *S. alterniflora*, ensuring sustainable coastal management in China and informing global invasive species strategies.

**Fig. 1:**
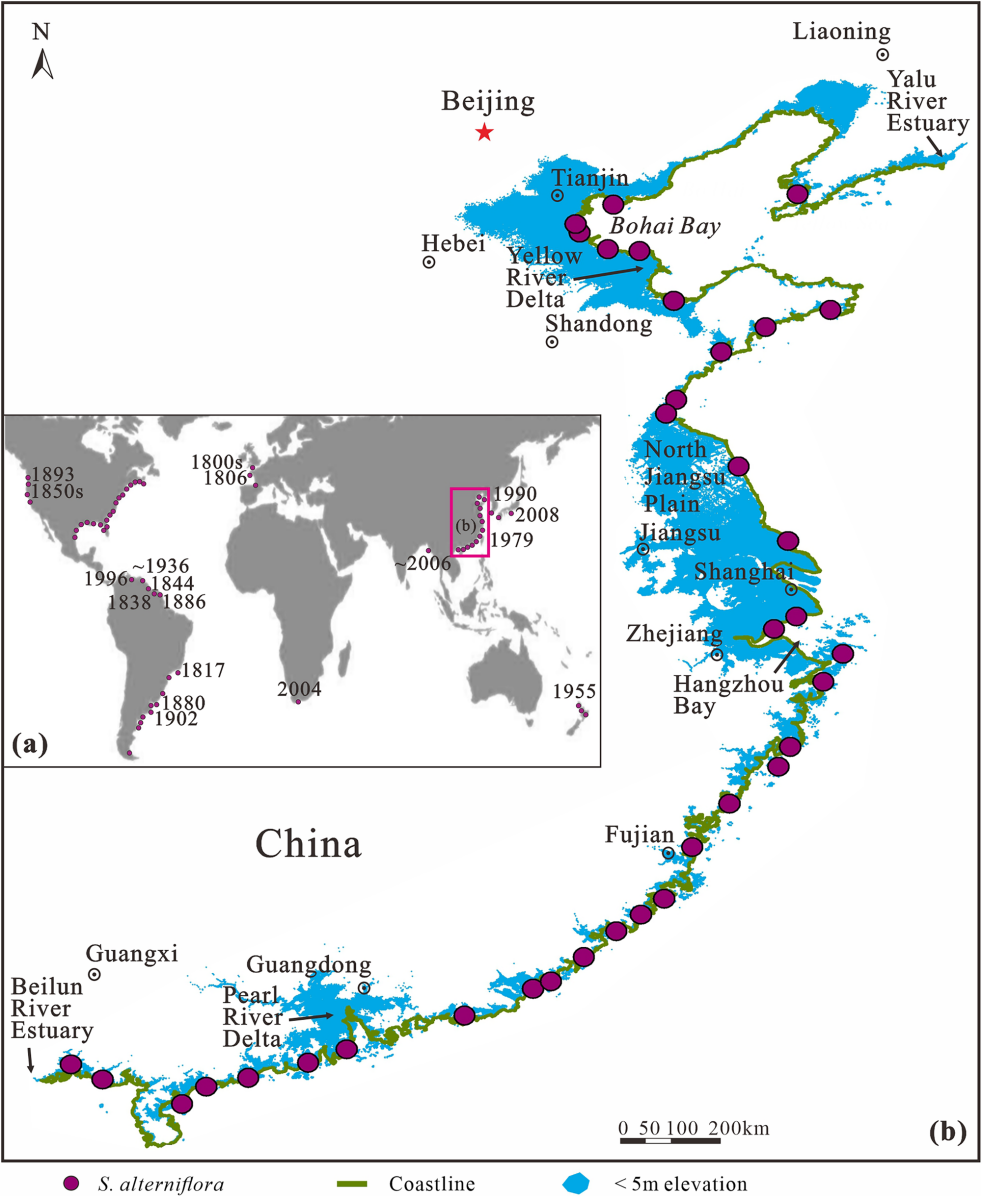
Global and China’s mainland coastal distribution of *S. alterniflora*. Distribution of *S. alterniflora* globally (**a**) and in China’s mainland (**b**). The numbers in (**a**) indicate the initial invasion time of *S. alterniflora*; purple dots in (**a**, **b**) represent *S. alterniflora*; the green line in (**b**) is the present coastline, and the blue shaded areas represent regions with an elevation below 5 m.

## Policy and management efforts

China’s response to 82,000-hectare invasion of *S. alterniflora* includes the *Special Action Plan for the Prevention and Control of S. alterniflora (2022−2025)*^7^, issued by multiple ministries to control the species by 2025 through a four-year eradication campaign. As of September 2024, over 80% of invaded areas were cleared using physical methods (e.g., manual uprooting, mechanical excavation, smothering), because they are regarded as faster speed and lower ecological risk compared to chemical, biological, or integrated controls (Supplementary S2). However, physical removal is costly, labor-intensive, and requires repeated interventions, with regrowth documented in Shandong, Shanghai, and Zhejiang.

Physical methods also cause substantial ecological disturbances, damaging benthic habitats and invertebrate communities. For instance, China Green Express reported that large-scale removal in Tianjin reduced populations of the vulnerable Relic Gull (*Ichthyaetus relictus*), an IUCN Red List species. Sediment disturbance releases carbon-, nitrogen- and sulfur-containing gases (e.g., CH_4_, NH_3_, H_2_S), compromising habitat quality (Supplementary S2)^8^. Moreover, without post-removal management, such as native plant restoration, regrowth undermines long-term effectiveness. The adverse impacts of invasive species control measures have received minimal attention^9^.

China’s 18,000-km mainland coastline, spanning 22 degrees of latitude, divides at Hangzhou Bay into northern tidal flats and southern mangroves with distinct landscape (Supplementary S3). In the south, *S. alterniflora* directly competes with native mangroves (mainly *Avicennia marina*, *Kandelia obovata*, and *Aegiceras corniculatum*) in the intertidal zone, threatening regional biodiversity and ecosystem function. In contrast, the northern coast consists largely of unvegetated tidal flats and saltmarshes dominated by common reed (*Phragmites australis*) and seablite (*Suaeda salsa*), species less capable of colonizing the extensive areas lying below the high tide line. In these areas, *S. alterniflora* often occupies otherwise barren flats, with minimal competition from native flora. Therefore, its removal seldom promotes the recovery of native saltmarsh vegetation. Calls for complete eradication^10^ overlook these regional differences, risking ecological nuance and potential benefits, such as coastal protection^11,12^ in the northern areas, which becomes emerging under the background of global warming. The Action Plan’s one-size-fits-all approach fails to address these variations, incurring high costs and unintended consequences. Science-driven, adaptive policies are urgently needed to balance the ecological risks and benefits of *S. alterniflora*, ensuring sustainable coastal management.

## Dual impacts of *S. alterniflora*

As a well-known invasive species, *S. alterniflora* can disrupt local ecosystems through three primary mechanisms: a) competitive displacement of native plants *via* rapid clonal expansion, leading to the monopolization of intertidal zones and reduced plant diversity^13,14^; b) habitat modification *via* dense root mats that alter sediment dynamics and reduce tidal flat accessibility for migratory shorebirds^15,16^; and c) trophic cascade effects that restructure benthic invertebrate communities and diminish coastal fishery productivity^17,18^. Accordingly, *S. alterniflora* removal is often necessary to mitigate these impacts. However, its ecological consequences are not uniform and vary considerably across different coastal regions.

The primary ecological concern associated with *S. alterniflora* lies in its potential to encroach upon extensive bare tidal flats that serve as critical foraging grounds for migratory shorebirds. However, its distribution is restricted to the upper tidal flat (i.e. areas with < 40% submergence probability), leaving mid- and lower tidal zones as bare mudflats^19^. Targeted mowing to maintain patchy distributions could balance *S. alterniflora*’s coastal functions with shorebird habitat needs.

Despite these risks, *S. alterniflora* offers ecological and economic benefits. Field surveys show higher shellfish biomass near the *S. alterniflora* stands, potentially enhancing food availability for shorebirds (Supplementary S4). Meanwhile, several bird species, such as Mallard (*Anas platyrhynchos*), Eastern Spot-billed Duck (*Anas zonorhyncha*), Pallas’s bunting (*Emberiza pallasi*), and Reed parrotbill (*Paradoxornis heudei*)^20,21^, have been observed roosting within *S. alterniflora* stands during winter. Additionally, despite ongoing debate, evidence indicates that the proliferation of *S. alterniflora* enhances the ecosystem’s carbon and nitrogen storage capabilities^5^. The ongoing warming climate may further increase its carbon sequestration potential by stimulating physiological activities^6^.

Over the past two decades, our field investigations along the Bohai Bay coast have documented extensive oyster colonization within *S. alterniflora* stands accompanied by a marked increase in biomass. Biodiversity in the symbiotic area of *S. alterniflora* and oysters was observed slightly higher, and the total biomass is nearly 10 times ( ~ 1.65 kg/m^2^) higher than that in the adjacent bare flat (Supplementary S4). Our 2023−2024 fieldwork also identified new shell accumulations (Supplementary S4) in and around the *S. alterniflora* stand, either forming shelly bars or attaching to seawalls at high tidal level, which indicate potential initiation of new chenier formations. This also means that the chenier plains of Bohai Bay^22,23^ remain geomorphologically and ecologically active. Similar trends are observed along the Jiangsu coast, where *S. alterniflora* seems to aid the recovery of native oyster reefs by improving substrate stability and habitat conditions.

*S. alterniflora* also exhibits strong capabilities in promoting sediment accretion and enhancing coastal protection. With accelerating sea-level rise, increased coastal erosion, and ongoing land subsidence, strengthening the resilience of muddy coasts has become an urgent global priority. In Bohai Bay, sedimentation rates within colonized zones reach 3−4 cm/year, compared to <1 cm/year in uncolonized areas. This vertical accretion elevates the tidal flat surface, thereby improving the protective function of the seaward zones and alleviating wave pressure on landward seawalls. Similar sedimentation-enhancing effects have also been reported along the Jiangsu coast (Supplementary S3).

These biological-geological processes hold considerable practical value for modern coastal defense, particularly given the limitations of relying solely on hard infrastructure such as seawalls^24^. As warming above 1.5°C is expected to drive widespread retreat of coastal habitats^25^, the role of *S. alterniflora* in maintaining intertidal elevation and ecosystem stability warrants serious consideration. Therefore, the adaptive management of *S. alterniflora* should carefully weigh its positive and negative effects, which vary across locations. A uniform removal policy is thus inappropriate. Instead, there is an urgent need for scientifically grounded, nature-based and site-specific management policies.

A uniform eradication policy ignores these regional benefits and risks ecological disruptions. Adaptive, science-driven management—leveraging *Spartina*’s 82,000-hectare biomass (*ca*. 1.13 ~ 1.73×10^6^ ton/year)^26^, and sediment benefits while controlling its spread—is critical for sustainable coastal protection and global invasive species strategies.

## Towards a balanced adaptive management

The management of *S. alterniflora* requires a nuanced, science-driven strategy that acknowledges both its ecological risks and potential benefits. Rather than pursuing complete eradication, effective control should prioritize location-specific adaptive strategies, informed by robust assessments of site conditions, ecological trade-offs and long-term resilience goals. Ecological niche studies (Supplementary S5) suggest that the seaward expansion of *S. alterniflora* across tidal flats can be regulated, although the spatial representativeness of existing case studies and uncertainties regarding long-term ecosystem responses across heterogeneous coastal environments should be acknowledged (Supplementary S6). In this context, national and regional policies should shift from blanket eradication toward integrated, ecologically based management. Interventions must be implemented cautiously to avoid ineffective investments and unintended ecological consequences. We propose four evidence-based reforms:

i. Enhance Monitoring and Research: Establish a national *S. alterniflora* monitoring network using satellite imagery and ecological niche modeling to track spatial dynamics and quantify trade-offs (e.g., biodiversity loss vs. carbon storage). Fund research to tailor interventions to regional conditions, such as northern tidal flats versus southern mangroves, reducing wasteful spending.
ii. Optimize Northern Coast Management: In northern tidal flats (e.g., Bohai Bay, Jiangsu), control *S. alterniflora*’s expansion through targeted mowing and partial retention to harness sediment accretion (3–4 cm/year) and oyster habitat benefits (1.65 kg/m² biomass). Pilot integrated physical and biological controls to test scalability and cost-effectiveness.
iii. Adopt Phased Ecological Interventions: In southern mangroves, implement gradual removal using integrated methods, such as native plant restoration, to minimize benthic disruption and greenhouse gas emissions. Phased approaches ensure ecosystem recovery while preserving biodiversity.
iv. Explore Sustainable Utilization: Leverage *S. alterniflora*’s ca.1.43 × 10^6^ tons of annual biomass for biofuel, feed, or salt-tolerant gene applications. Public-private partnerships can fund pilot projects, transforming *S. alterniflora* into a resource while reducing invasion pressure.

## Supplementary information

Supplementary information

## Data Availability

No datasets were generated or analysed during the current study.
