# Supplementary material for: Reforming Spartina alterniflora policies for sustainable coastal resilience: an evidence-based adaptive strategy
Source: NPJ Biodivers. 2026 Aug 5;5:25. doi: 10.1038/s44185-026-00149-2 (PMC13443920; doi:10.1038/s44185-026-00149-2)
Supplement: Supplementary file 1 [file 44185_2026_149_MOESM1_ESM.pdf]

Supplementary Information

Reforming *Spartina alterniflora* Policies for Sustainable Coastal Resilience: An evidence-based adaptive strategy

Fu Wang[1,2]✉, Hong Wang[1,2], Qian Yu[3]✉, Peng Yang[1,2], Shu Gao[3], Yunzhuang Hu[1,2], Xueqiang Lu[4,5]✉

[1] *Tianjin Center (North China Center of Geoscience Innovation), CGS Key Laboratory of Coast Geo-Environment, China Geological Survey (CGS), Tianjin 300170, China*

[2] *Tianjin Key Laboratory of Coast Geological Processes and Environmental Safety, Tianjin 300170, China*

[3] *Key Laboratory of Coast and Island Development of the Ministry of Education, School of Geography and Ocean Science, Nanjing University, Nanjing 210023, China*

[4] *Tianjin Key Laboratory of Environmental Technology for Complex Trans-Media Pollution and Tianjin International Joint Research Center for Environmental Biogeochemical Technology, Tianjin 300350, China*

[5] *College of Environmental Science and Engineering, Nankai University, Tianjin 300350, China*

✉ Corresponding authors: tjwangfu@163.com; qianyu.nju@gmail.com; luxq@nankai.edu.cn

Content

S1. *S. alterniflora* on global coasts ... 1
Fig. S1. Global distribution and historical spread of *S. alterniflora* since the 19th century. ... 1
Fig. S2. Representative habitats of *S. alterniflora* along the mainland coast of China. ... 1
S2. Control of *S. alterniflora* and its negative effects ... 2
(1) Four categories of *S. alterniflora* control methods ... 2
Fig. S3. Physical control of *S. alterniflora* in Binhai New Area, Tianjin (left) and Qingdao coast, Shandong Province (right) (Figure legend[2]), China ... 2
Fig. S4. Chemical control of *S. alterniflora* in San Francisco Bay, USA (Figure legend[3]). ... 3
Fig. S5. Replacement of S. alterniflora by mangroves along the Quanzhou coast, Fujian Province, China (Figure legend[4]). ... 3
Fig. S6. Replacement of hybrid *Spartina* in Cargill Marsh, San Francisco Estuary, USA. ... 4
(2) Control methods used in China and their negative ecological effects ... 4
S3. Uniform eradication policy for *S. alterniflora* may not be appropriate for China's diverse coastal settings ... 6
(1) China's coastal geomorphological context ... 6
(2) Potential role of *S. alterniflora* in coastal protection ... 6
Fig. S7. Mainland coast of China and major geomorphological coastal types. ... 7
S4. Newly observed phenomenon: Coexistence of *S. alterniflora* and oysters ... 9
(1) Association between oysters and *S. alterniflora* ... 9
Fig. S8. Coexistence of oysters and *S. alterniflora* on the coast of Bohai Bay, China. ... 9
Table S1. Density and community composition of macrobenthic organisms. ... 10
(2) Formation of shell accumulations and potential shelly cheniers ... 10
Fig. S9. Shell accumulation surrounding *S. alterniflora* stands. ... 11
S5. Seaward expansion *S. alterniflora* on tidal flat is environmentally constrained ... 12
Fig. S10. Remote sensing images of *S. alterniflora* in central Bohai Bay coast, Tianjin, China. ... 12
Fig. S11. Elevation measurements of tidal flats using GPS-RTK and airborne LiDAR. ... 13
Fig. S12. Distribution and elevation range of *S. alterniflora* on the central Bohai Bay coast. ... 14
S6. Limitations and Uncertainties ... 15
Figure legends ... 16
References ... 16

S1. *S. alterniflora* on global coasts

S. alterniflora originated from the east coast of North America and the Gulf of Mexico, and began spreading to other regions in the 1800s, primarily through human activities. It was introduced to Europe as early as 1806 (1), to South America in 1817 (2), to the west coast of North America in the 1850s (3), to Australia (4) and New Zealand in 1955 (5), to China in 1979 (6), to South Korea in 1990 (7), to South Africa in 2004 (8), to Bangladesh before 2006 (9), to Japan in 2010 (10) (Fig. S1).

At present, *S. alterniflora* occurs along coasts across Asia, Europe, the Americas, Africa, Australia, and New Zealand. Its rapid expansion and strong competitive ability have made it a globally recognized invasive species. Owing to its high productivity and capacity to modify sediment dynamics and habitat structure, it is widely regarded as a classic example of an ecosystem engineer (11).

S. alterniflora was first introduced to the coast of Fujian province, China in 1979 for ecological engineering purposes. Since then, it has spread across all coastal provinces of mainland China to varying degrees, with rapid expansion in some regions. It now covers more than 66,000 hectares along China's mainland coastline (Fig. S2). In early 2003, *S. alterniflora* was officially designated as a coastal invasive alien species in China, and as of January 2023, it remains classified as such on the "Key Management List of Invasive Alien Species"(12). *S. alterniflora* has attracted widespread attention from scholars worldwide. In China, for example, the annual number of publications on *S. alterniflora* now exceeds 100 articles and continues to increase.

On December 5, 2022, a consortium of Chinese governmental bodies jointly issued the "Special Action Plan for the Prevention and Control of *S. alterniflora* (2022–2025)" (13), which seeks to bring the species under effective control by 2025 through a dedicated four-year removal effort.

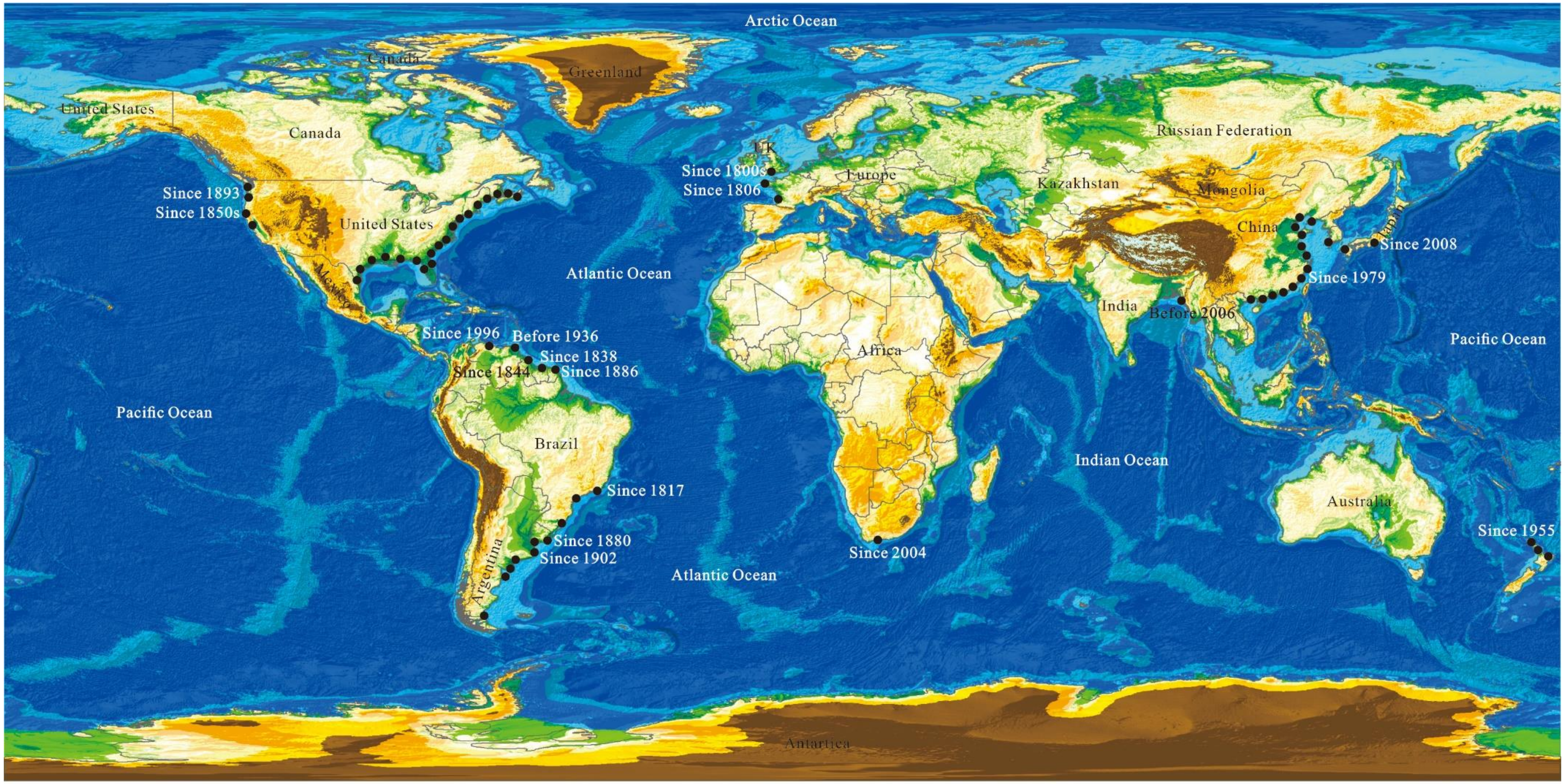

● *S. alterniflora* "Since 1902"—initial invasion time of *S. alterniflora*

Fig. S1. Global distribution and historical spread of *S. alterniflora* since the 19th century.
The black dots represent *S. alterniflora*, and the numbers (such as "Since 1902") indicate the initial invasion time of *S. alterniflora*.

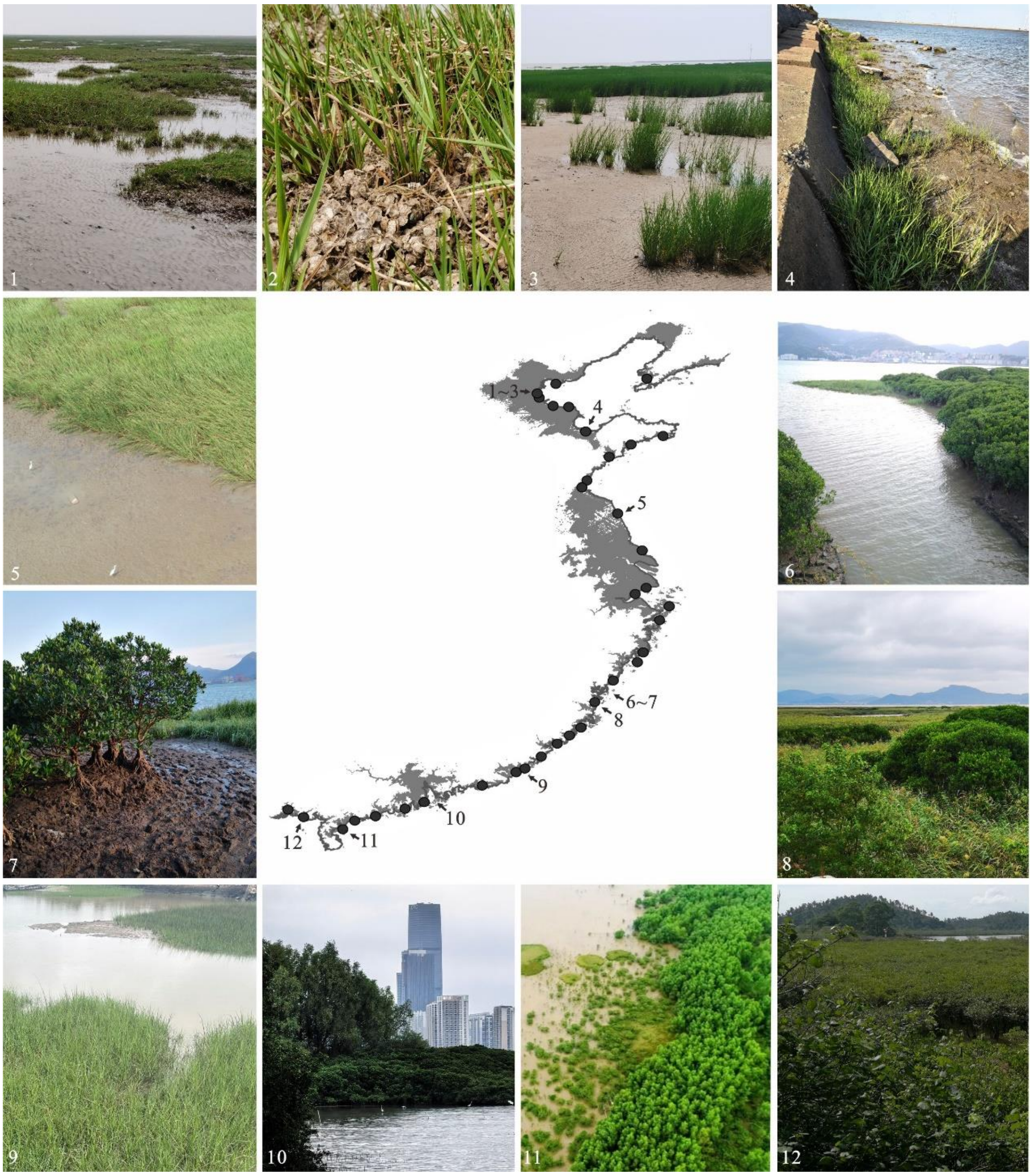

Fig. S2. Representative habitats of *S. alterniflora* along the mainland coast of China.

Photos 1~3: Mixed oyster reefs and *S. alterniflora* habitats; Photo 4: *S. alterniflora* along a coastal dyke; Photo 5: *S. alterniflora* habitat; Photos 6 and 7: Coexistence of mangrove and *S. alterniflora*; Photo 8: Restored estuarine wetland invaded by *S. alterniflora*; Photo 9: Reinvasion of *S. alterniflora*; Photo 10: Mangrove ecosystem near a megacity; Photo 11: Mangrove ecosystem invaded by *S. alterniflora* (**Figure legend**[1]); Photo 12: Mangrove ecosystem.

S2. Control of *S. alterniflora* and its negative effects

Efforts to control *S. alterniflora* and other *S. species* have been reported along the coasts of China, the west coast of the United States, estuaries in Northern Ireland (UK), the Atlantic coast of Spain, the Great Brak River estuary in South Africa, the southern coast of Australia, and the northern coast of New Zealand. Among these regions, the largest-scale control programs have been undertaken in China, the United States and western Canada. Control strategies generally include physical, chemical, biological, and integrated approaches.

(1) Four categories of *S. alterniflora* control methods

① Physical control

Mechanical or manual removal methods, including uprooting, cutting, tillage, and flooding, are commonly used to suppress the photosynthesis or respiration of *S. alterniflora*, thereby limiting its growth and reproduction **(Fig. S3)**. These approaches generally avoid chemical pollution and can produce rapid short-term effects. However, they are labor-intensive and costly, require long treatment cycles, and inevitably cause substantial disturbance to intertidal environments, particularly to sediment structure and benthic communities, which may require post-treatment ecological restoration. Physical control has been primarily applied along the coast of China and in several coastal regions of the United States.

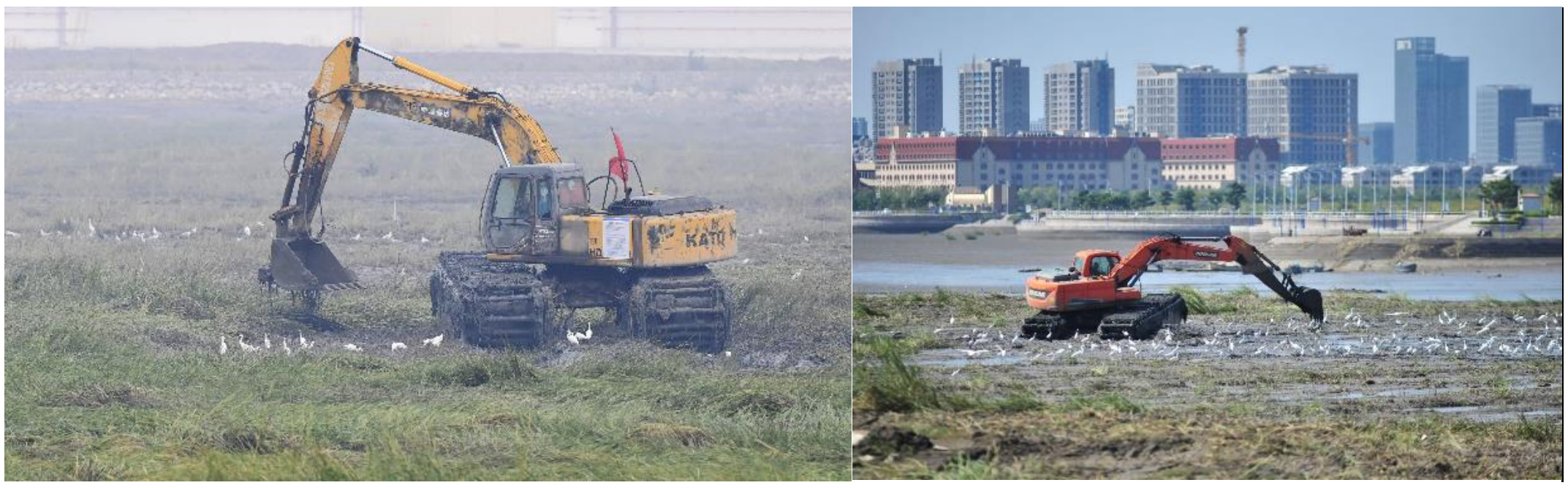

Fig. S3. Physical control of *S. alterniflora* in Binhai New Area, Tianjin (left) and Qingdao coast, Shandong Province (right) (**Figure legend**[2]), China

② Chemical control

Chemical control involves the application of various herbicides to suppress the growth and reproduction of *S. alterniflora* **(Fig. S4)**. While this method is convenient and relatively low cost, its effectiveness is strongly influenced by environmental factors

such as wind, tide cycle, and the wax layer covering plant stems and leaves. Moreover, the use of chemicals may have adverse effects on sediment quality, native vegetation, and benthic organisms. Chemical control has been applied in multiple regions worldwide.

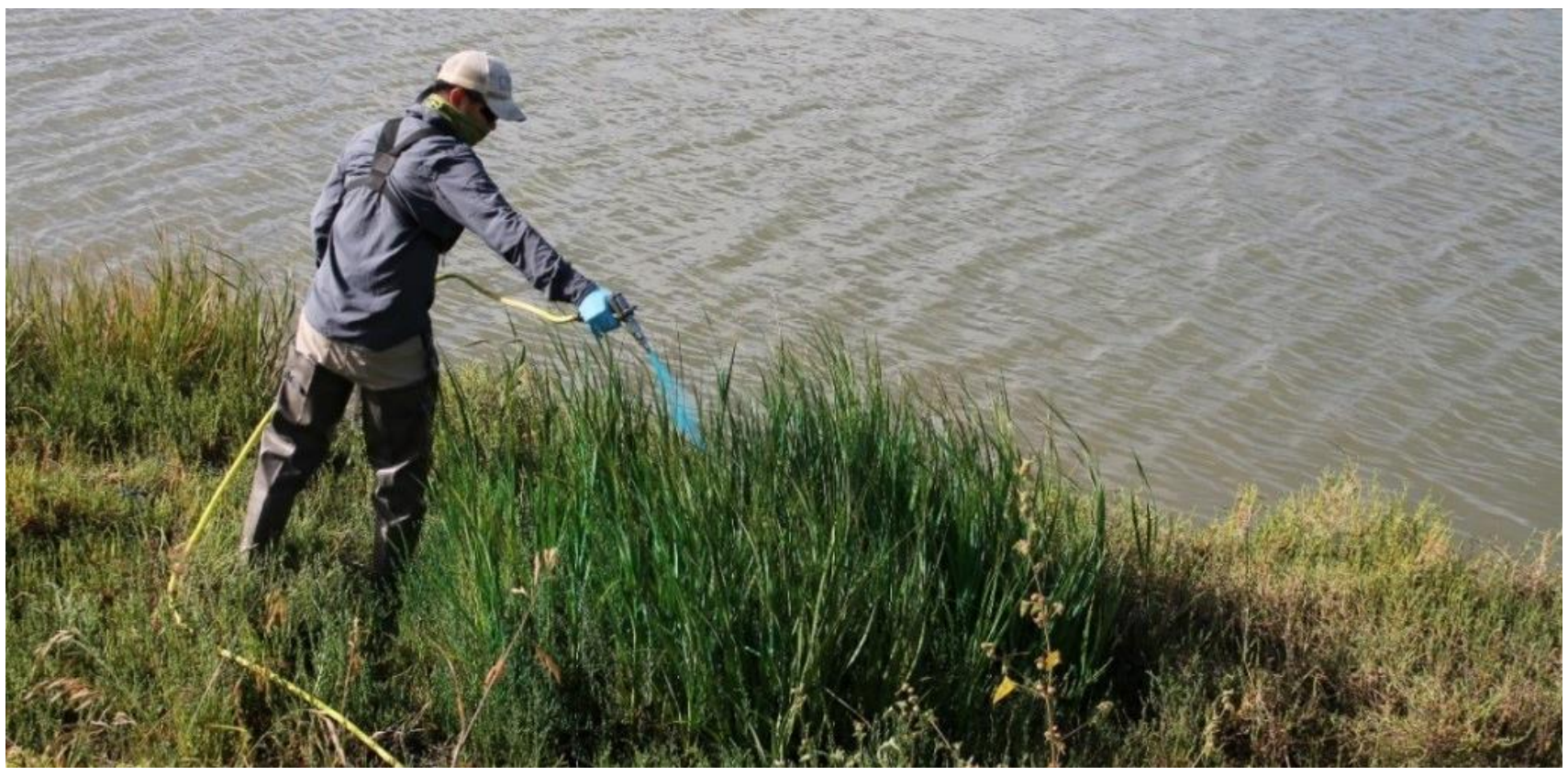

Fig. S4. Chemical control of *S. alterniflora* in San Francisco Bay, USA (**Figure legend**[3]).

③ Biological control

Biological control utilizes one or more organisms to suppress the growth and reproduction of *S. alterniflora*, based on the ecological principles such as predator-prey relationships and interspecific competition **(Figs. S5 and S6)**. Alternatively, native plant species with ecological or economic value, such as common reed (*Phragmites australis*) and mangroves, may be introduced to replace *S. alterniflora*, through the natural processes of plant community succession. However, selecting suitable replacement species remains challenging, and rigorous ecological risk assessment is necessary to avoid unintended impacts on native ecosystems.

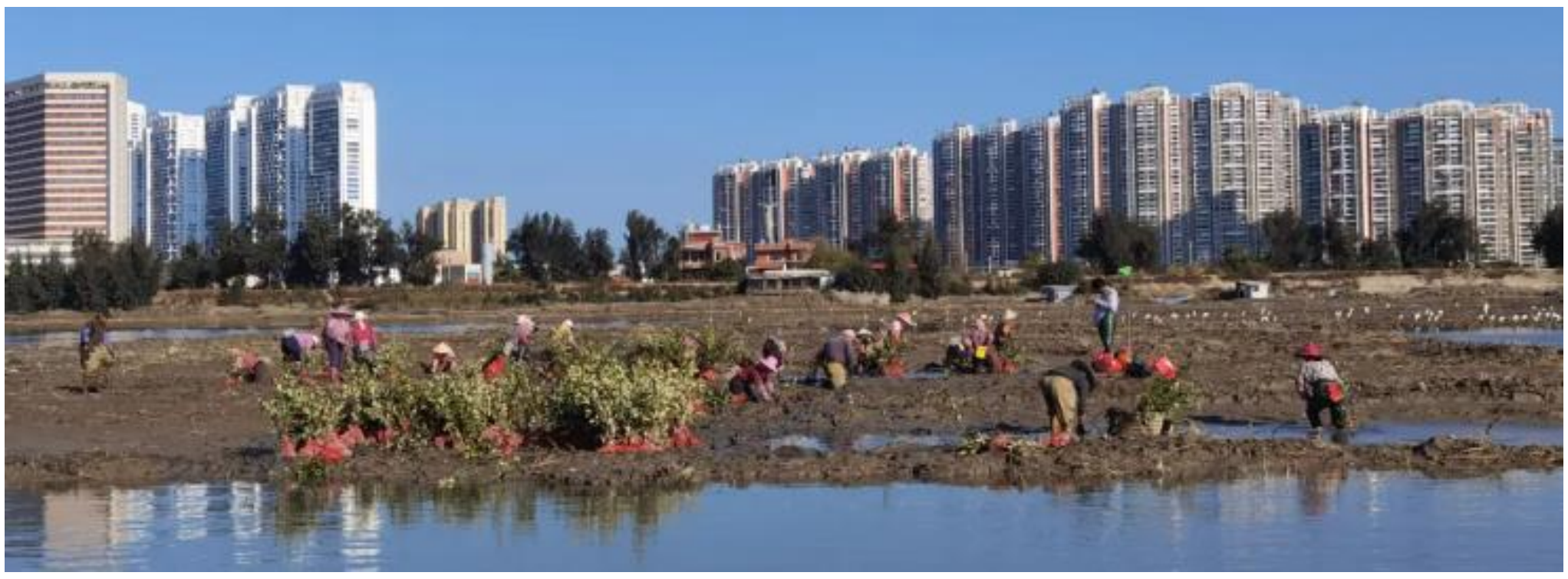

Fig. S5. Replacement of S. alterniflora by mangroves along the Quanzhou coast, Fujian Province, China (**Figure legend**[4]).

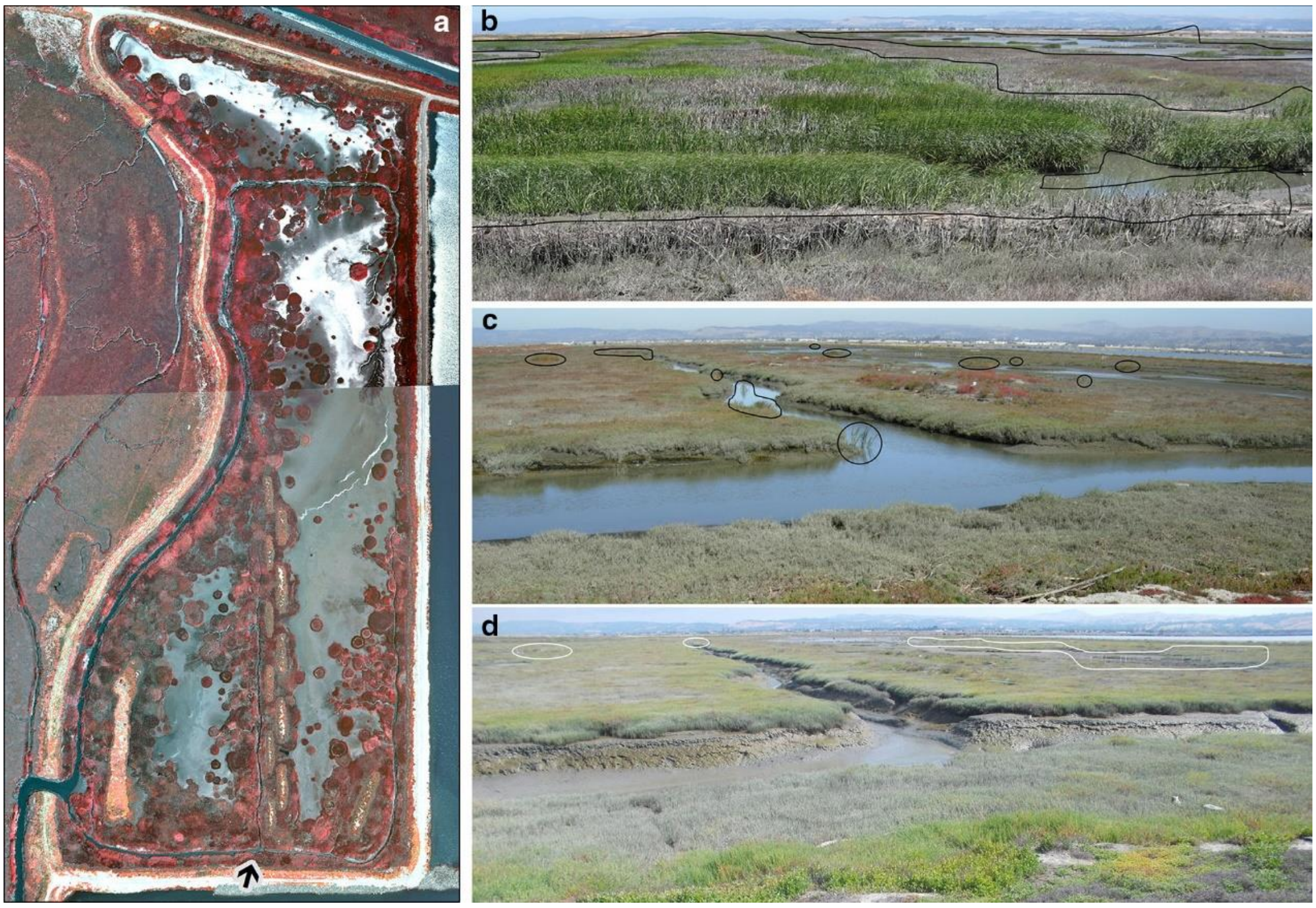

Fig. S6. Replacement of hybrid *Spartina* in Cargill Marsh, San Francisco Estuary, USA.
Photo a: In 2005, a 21−hectare field contained 9 hectares of hybrid *Spartina*, with circular patches of individual hybrid plants clearly visible. The arrows in (a) indicate the location of time-series photos shown in (b-d). Photo b: In 2006, hybrid *Spartina* (outlined in black) had filled the canal and became the dominant species in the area. Photo c: In 2010, hybrid *Spartina* had declined to sparse patches. Photo D: In 2014, less than 1 m2 of hybrid *Spartina* remained at the site, largely replaced by native *Spartina foliosa* and *Grindelia stricta* (outlined in white) (14).

④ Integrated control

Integrated control combines multiple approaches to achieve enhanced efficacy in suppressing the growth and reproduction of *S. alterniflora*. For instance, physical methods−such as cutting to remove aboveground biomass−can be applied first, followed by flooding to induce anaerobic conditions that decompose belowground tissues. Herbicides may then be used selectively to eliminate residual plants.

(2) Control methods used in China and their negative ecological effects

In China, the predominant methods are physical and biological control (15,16). In recent years, large-scale coastal restoration projects have extensively applied physical removal methods to manage *S. alterniflora*. In addition, projects in the United States, South Africa, the Netherlands, and Canada have adopted integrated strategies combining physical, biological, and chemical control approaches (8,17-24).

While physical control can achieve rapid short-term reduction in *S. alterniflora* coverage, repeated interventions are often required, resulting in high economic coasts

and logistical challenges. Without sustained long-term management, reinvasion frequently occurs. Monitoring studies from Dongying (Shandong Province) (21), Chongming Dongtan (Shanghai) (18), and Xiangshan Port (Zhejiang Province) (20) have documented varying degrees of *S. alterniflora* regrowth following physical removal.

S. alterniflora can enhance primary productivity (25) and increase sediment carbon, nitrogen, and sulfur storage (26,27). However, physical disturbance may accelerate the release of stored C, N, and S when previously buried sediments are exposed to oxygen. More importantly, intensive physical removal can damage benthic habitats and reduce benthic biomass (28). For example, China Green Express reported a marked decline in population of relic gull (*Larus relictus*), a protected species, following the physical eradication of *S. alterniflora* along the northern coast of Tianjin, China (29).

Based on current project estimates, complete eradication of *S. alterniflora* across China would cost about 9 billion RMB (1.26 billion USD). Rather than allocating the entire budget solely to eradication, a portion of these resources could support a coordinated national research program to better evaluate the species' ecological effects across diverse coastal settings. Given the substantial regional variability in reported impacts, such research may help identify management approaches that mitigate negative effects while explore potential pathways to use *S. alterniflora* as a functional component in coastal stabilization and ecosystem restoration.

S3. Uniform eradication policy for *S. alterniflora* may not be appropriate for China's diverse coastal settings

(1) China's coastal geomorphological context

China's coastline spans approximately 32,000 km, including about 18,000 km along the mainland coast, stretching from the Yalu River estuary in the north to the Beilun River estuary in the south (**Fig. S7**).

From north to south, the major structural units include Bohai Bay Basin, Yanshan, Luxi–Ludong Uplift, South Yellow Sea Basin, and Zhejiang-Fujian Uplift, adjacent to the Southern North China Basin and North Yellow Sea Basin. From a geological perspective, the mainland coastline can be broadly divided into two contrasting types: the subsiding muddy coast of northern China, extending approximately 6,000 km, and the uplifted bedrock and sandy coast of southern China, extending approximately 12,000 km. The transition between two coastal types occurs near Hangzhou Bay.

The most recent major marine transgression began in the early Holocene. During 6-7 ka BP, seawater inundated up to 80-100 km inland in regions such as Bohai Bay, the North Jiangsu Coast, and the Pearl River delta. During this period, the western Bohai Bay coast and the Jiangsu coast was transformed into intertidal to shallow marine environments. From the mid-Holocene to approximately 1850 CE, high suspended sediment loads delivered by the Yellow River and Yangtze River promoted delta progradation, gradually converting these shallow marine areas into coastal lowland. However, since approximately 1850 CE, a renewed phase of marine transgression has occurred due to land subsidence (30), sea-level rise (31,32), and reduced river sediment discharge (33). These processes have increased the vulnerability of large sections of China's muddy coasts to erosion (34) and coastal flooding (35,36).

(2) Potential role of *S. alterniflora* in coastal protection

As in many other regions worldwide, China's coastal zones are among the most economically developed and densely populated areas of the country. Coastal erosion and flooding pose risks to infrastructure, ecosystems, and sustainable development.

Previous studies indicate that more than 110,000 km^2 of coastal lowlands in China are highly vulnerable to sea-level rise, particularly along the northern coast between the Yalu River Estuary and Hangzhou Bay. This region corresponds to the subsiding muddy coast, encompassing the three bays of the Bohai Sea and the Jiangsu Coastal Plain, where the elevation of most areas is below +5m (**Fig. S7**). Unlike the uplifted southern

China coast, which is fringed by mangrove and other native vegetation, the tidal flats of the subsiding north China coast were initially bare and devoid of native vegetation. Therefore, as sea level continues to rise, it is urgent to protect the northern subsiding coast from erosion and to strengthen coastal dykes. *S. alterniflora*, originally introduced from the United States with the aim of stabilizing mudflats, reinforcing seawalls, and supporting land reclamation efforts, is now poised to fulfill these roles both now and in the future.

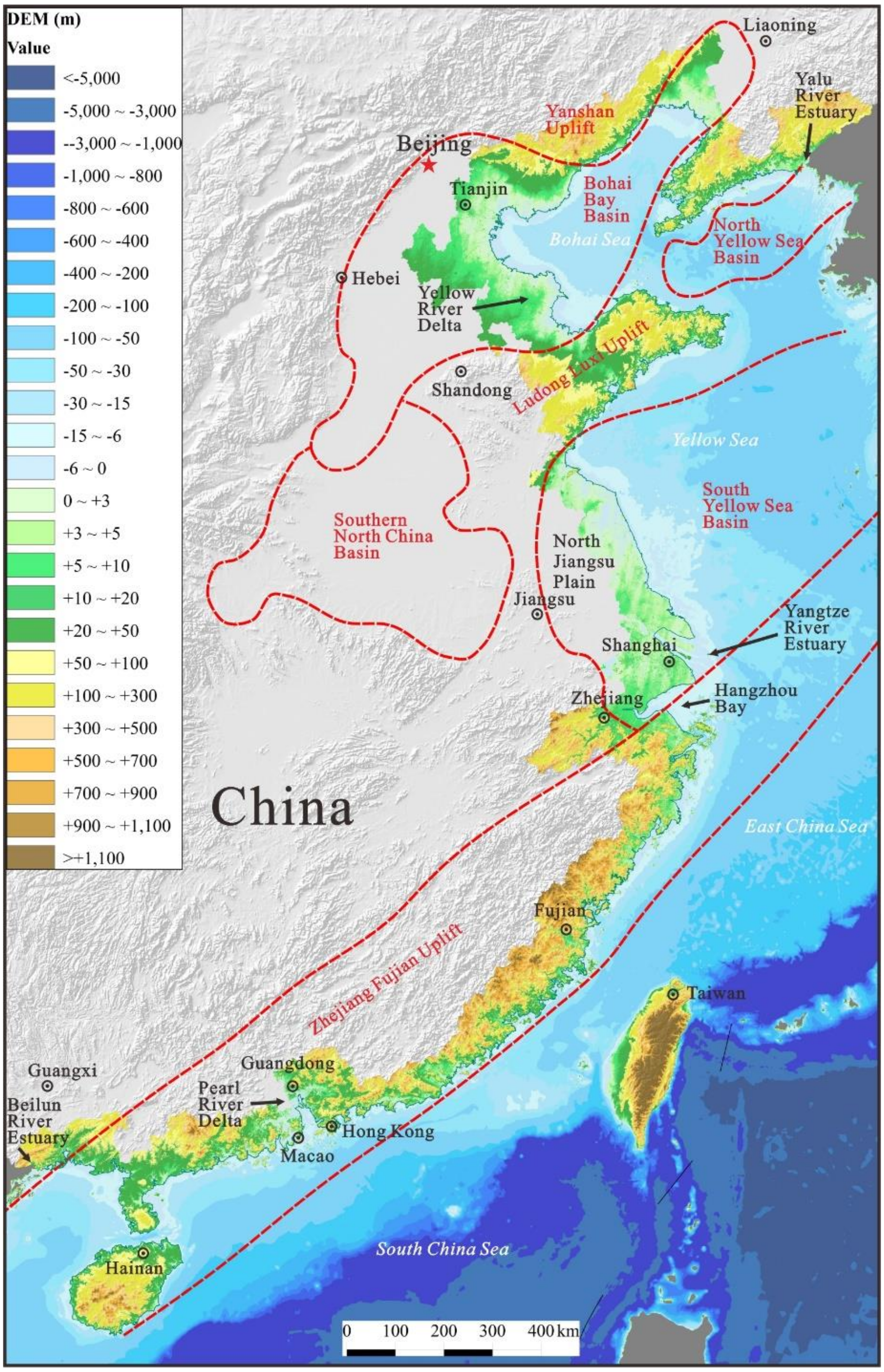

Fig. S7. Mainland coast of China and major geomorphological coastal types.

Sediment dating using ^{210}Pb and ^{137}Cs indicates that over the past century, the sedimentation rates in the upper intertidal zone of Bohai Bay ranged from 1.6 to 2.6 cm/year, while that in the lower intertidal zone is less than 1 cm/year (37,38). Since its expansion into the lower intertidal zone after 2010, *S. alterniflora* has markedly increased sedimentation rates. Monitoring data over the past 15 years show that areas colonized by *S. alterniflora* experienced average sedimentation rates of 3–4 cm/year, significantly higher than those in adjacent uncolonized areas. The resulting elevation gain of the tidal flat has enhanced the protective function of the seaward zones, thereby reducing pressure on landward seawalls. Similar sedimentation-enhancing effects of *S. alterniflora* have also been observed along the northern Jiangsu coast (39,40).

Current management policies emphasize complete eradication of *S. alterniflora* along China's coastline. However, given the diversity of coastal geomorphological settings, a more differentiated management strategy may be warranted. In particular, along portions of the subsiding muddy coasts of northern China, *S. alterniflora* may provide ecosystem functions related to sediment stabilization and shoreline protection. Therefore, adaptive management approaches that consider regional environmental conditions may be more effective than uniform eradication strategies.

S4. Newly observed phenomenon: Coexistence of *S. alterniflora* and oysters

(1) Association between oysters and *S. alterniflora*

Field observations indicate that *S. alterniflora* growing on the tidal flats can provide attachment substrate for oysters. Oyster shells are frequently observed attached to roots and lower stems of *S. alterniflora*, allowing colonization in soft muddy area where attachment would otherwise be difficult (**Fig. S8**).

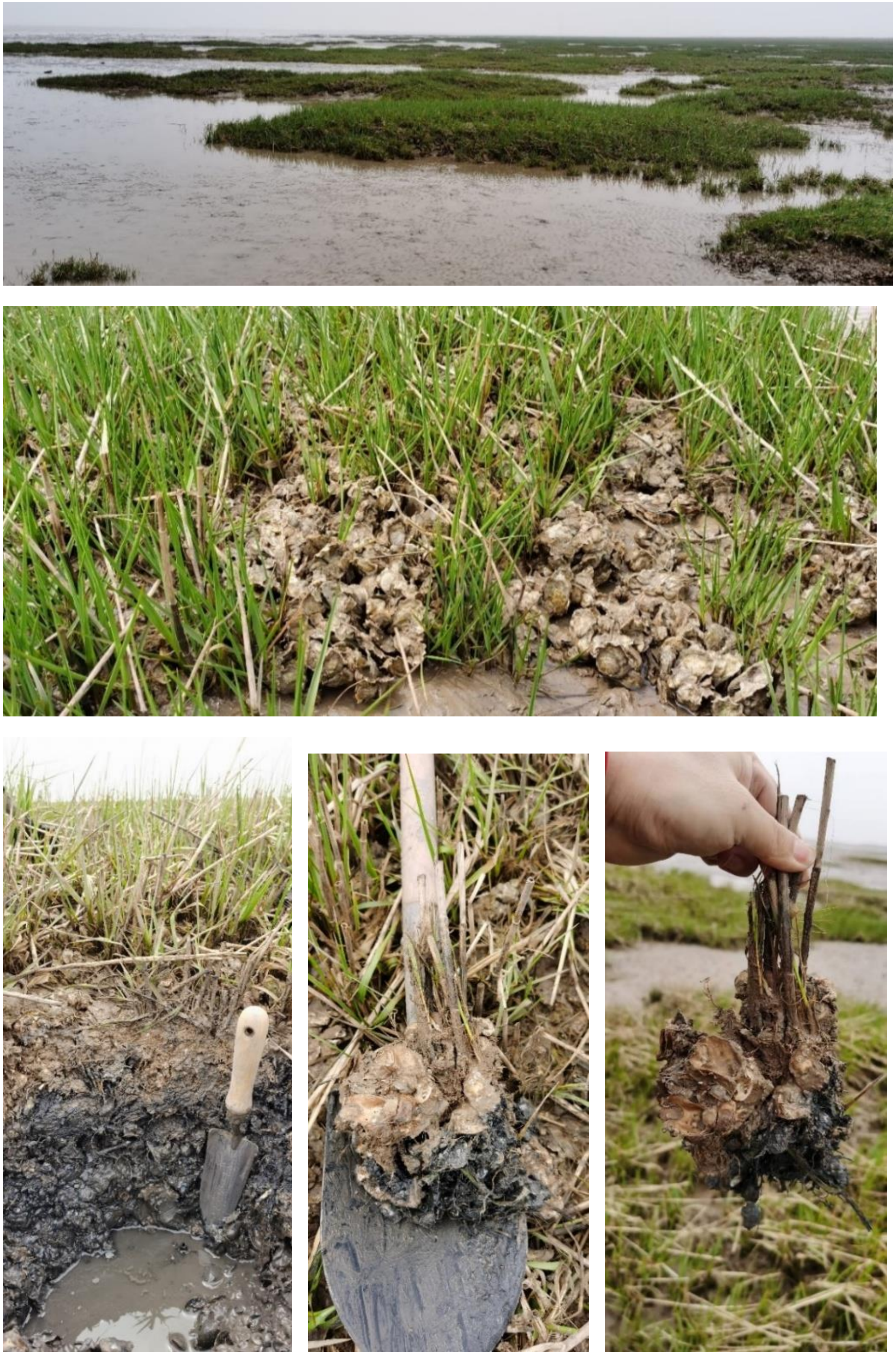

Fig. S8. Coexistence of oysters and *S. alterniflora* on the coast of Bohai Bay, China.

Due to this association, macrobenthic biomass within *S. alterniflora* habitats (site 01 in Table S1) in Bohai Bay is more than ten times higher than that observed in adjacent unvegetated tidal flats (site 02 in Table S1) and supratidal zones (site 03 in Table S1). These observations are consistent with previous studies conducted in Bohai Bay (41), the Yellow River Delta (42), and along the Jiangsu coast (43).

Table S1. Density and community composition of macrobenthic organisms.

Species		Site 01	Site 02	Site 03	Total
Annelida	density ind/m^2	110	50	15	175
	Biomass g/m^2	33.77	1.96	9.07	44.8
Spipuncula	density ind/m^2	5	0	0	5
	Biomass g/m^2	3.82	0	0	3.82
Mollusca	density ind/m^2	745	60	20	825
	Biomass g/m^2	1560.23	111.77	35.75	1707.75
Arthropoda	density ind/m^2	540	0	0	540
	Biomass g/m^2	41.3	0	0	41.3
Brachiopoda	density ind/m^2	20	10	0	30
	Biomass g/m^2	10.03	11.53	0	21.56
Others	density ind/m^2	5	0	0	5
	Biomass g/m^2	0.23	0	0	0.23
Total	density ind/m^2	1425	120	35	1580
	Biomass g/m^2	1649.38	125.26	44.82	1819.46

(2) Formation of shell accumulations and potential shelly cheniers

A large number of shells have been observed accumulating around *S. alterniflora* stands (**Fig. S9**). Over time, such shell accumulation processes may contribute to the formation of shelly cheniers.

Due to its rapid growth, high productivity, and salt tolerance, *S. alterniflora* is widely recognized as a typical ecosystem engineer (11). These observations suggest potential ecological functions beyond shoreline stabilization. The coexistence of oysters within *S. alterniflora* habitats indicates a coupled “plant + animal” coastal stabilization mechanism. In addition, field surveys indicate increased shellfish biomass in areas adjacent to *S. alterniflora* habitats, which may provide enhanced food resources for shorebirds (44-48).

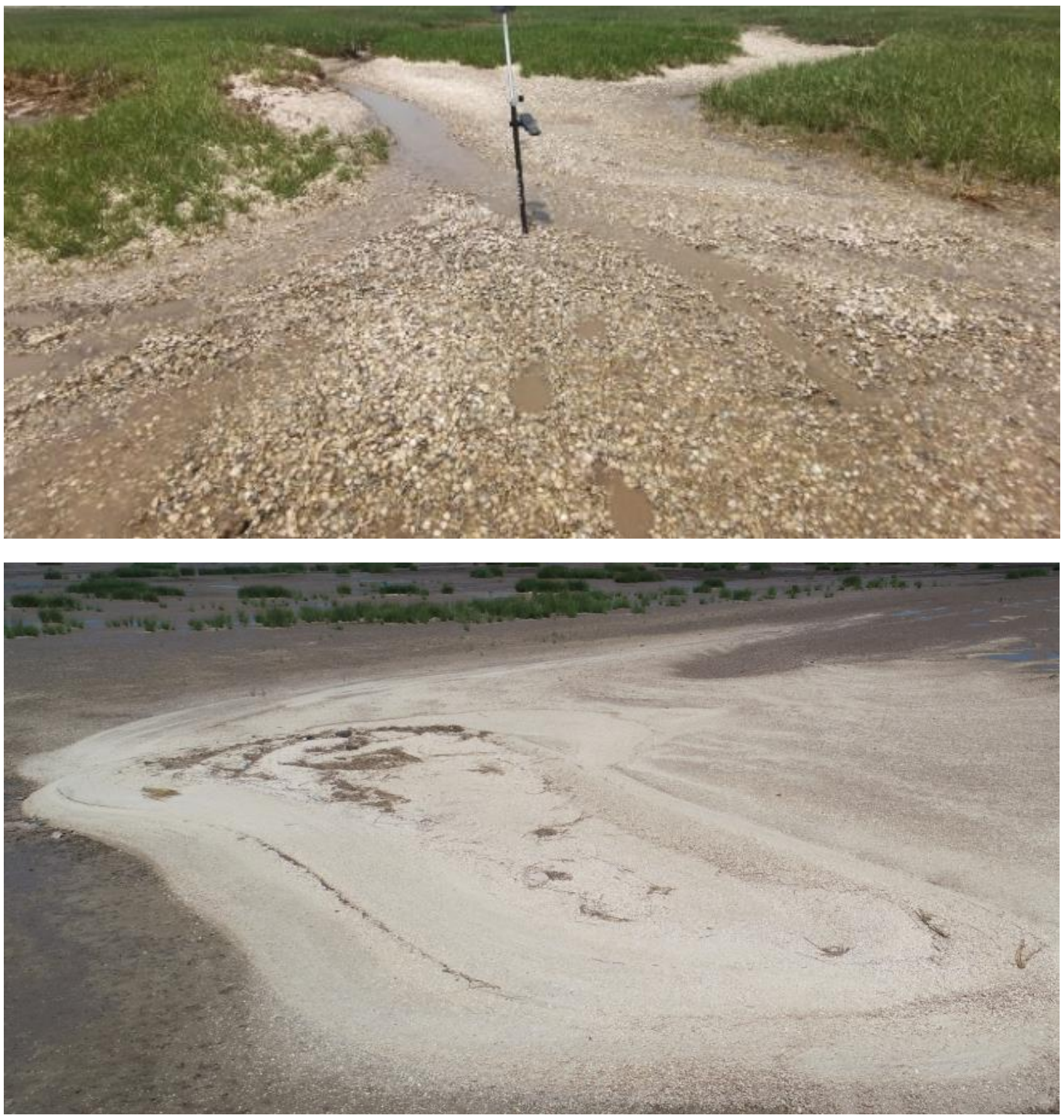

Fig. S9. Shell accumulation surrounding *S. alterniflora* stands.

These findings suggest that, in certain environmental contexts, management approaches integrating ecological functions of *S. alterniflora* may warrant further investigation alongside conventional eradication strategies.

S5. Seaward expansion *S. alterniflora* on tidal flat is environmentally constrained

To evaluate whether *S. alterniflora* expands indefinitely on tidal flats under natural conditions, two representative coastal regions were selected as a case study area: the central Bohai Bay coast (Tianjin) and the Yancheng coast (Jiangsu Province). Remote sensing imagery obtained from Google Earth (**Fig. S10**) was used to track temporal changes in *S. alterniflora* distribution, while airborne LiDAR and GPS-RTK surveys (**Fig. S11**) were used to determine elevation ranges of its seaward boundary.

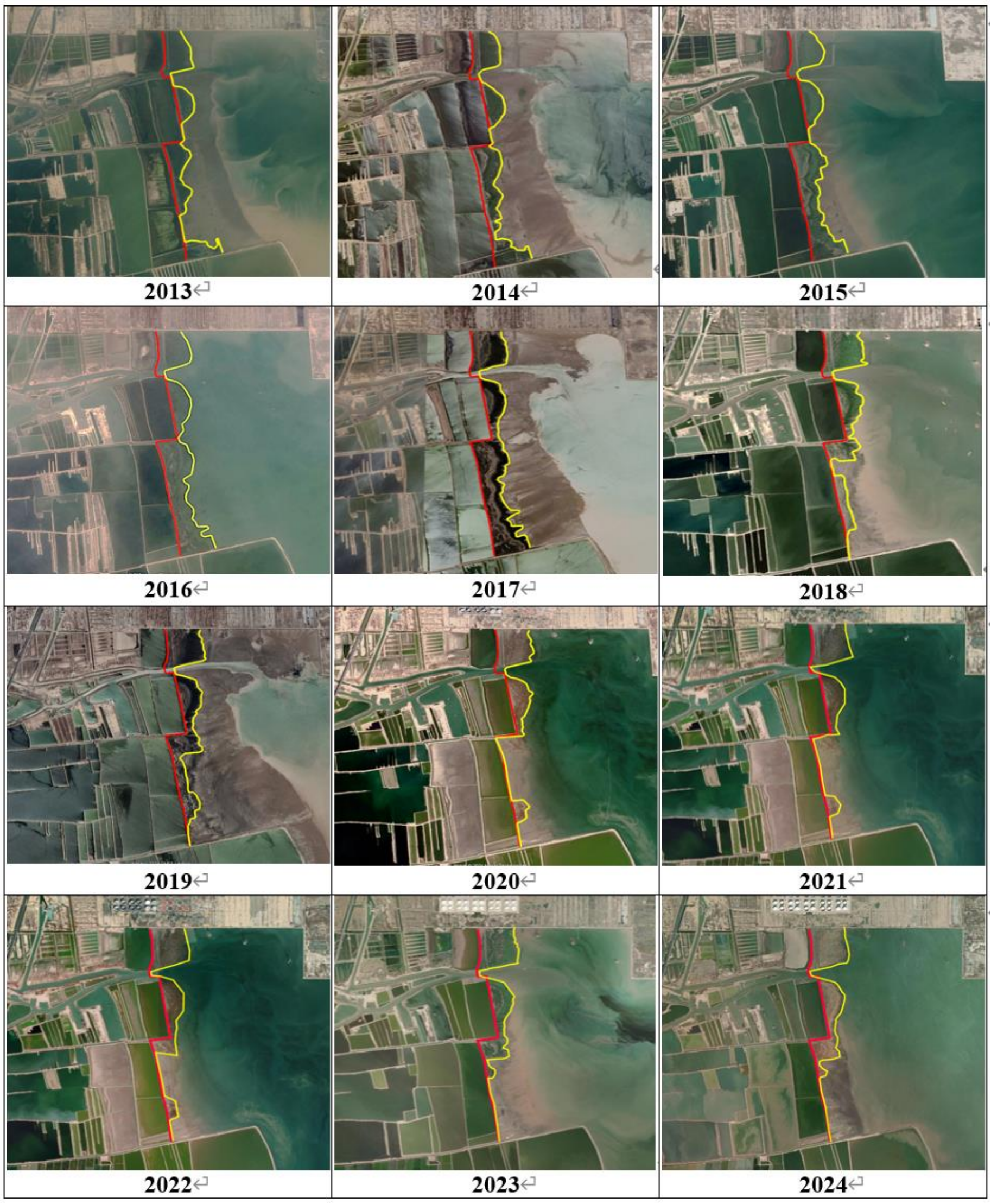

Fig. S10. Remote sensing images of *S. alterniflora* in central Bohai Bay coast, Tianjin, China.

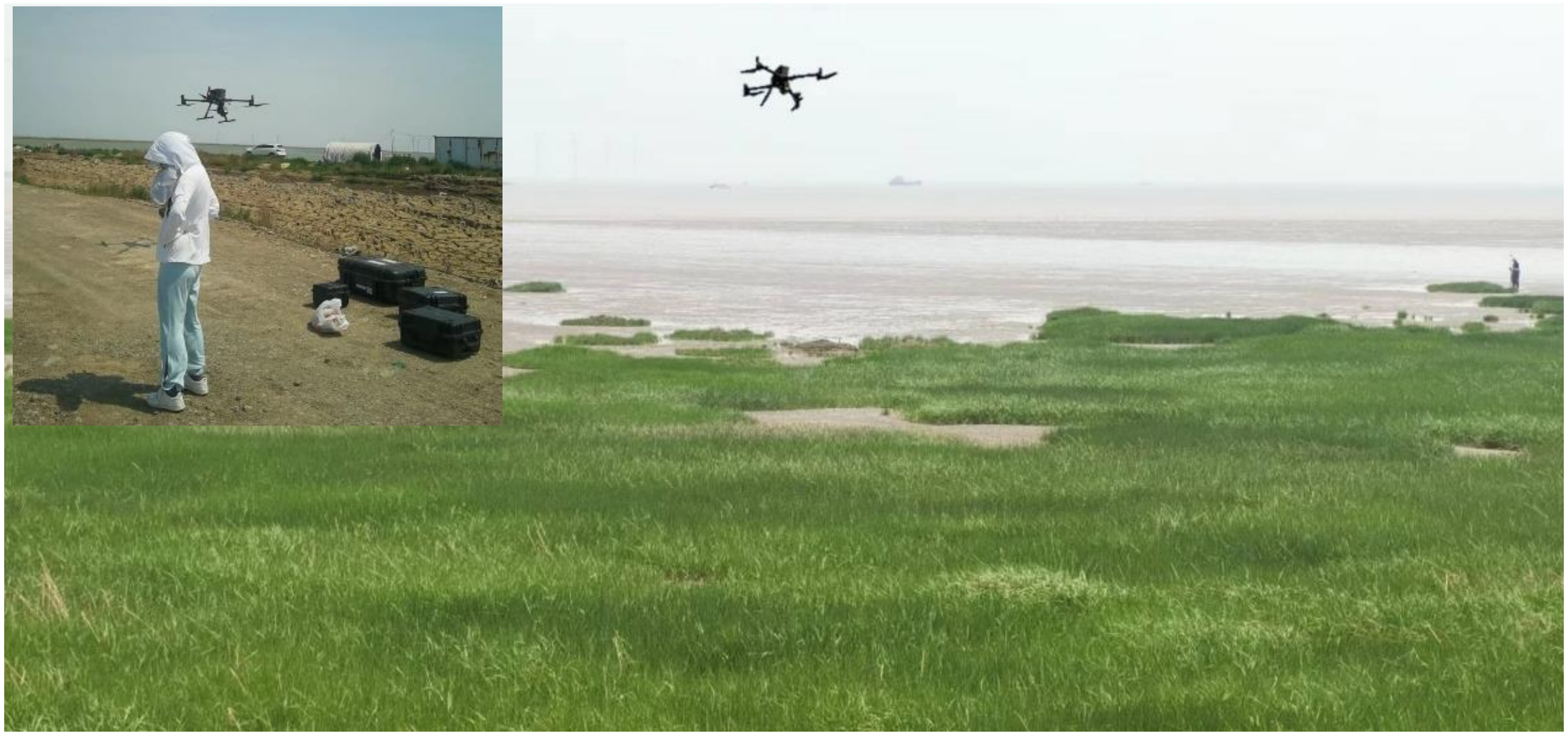

Fig. S11. Elevation measurements of tidal flats using GPS-RTK and airborne LiDAR.

Along the central Bohai Bay coast, as in many other regions of China (49), natural muddy shorelines have largely been replaced by engineered coastal structures, limiting landward expansion of *S. alterniflora*. However, the seaward boundary exhibits dynamic but spatially constrained fluctuations.

Between 2013 and 2017, the seaward boundary expanded in certain southern areas but was subsequently removed in 2018 due to management interventions. In northern areas without direct human disturbance, the seaward boundary shows natural oscillations within a relatively narrow zone along the outer margin of the main *S. alterniflora* distribution belt. A similar pattern has been observed along the Yancheng coast of Jiangsu Province.

Airborne LiDAR and GPS-RTK measurements indicate that *S. alterniflora* primarily occurs at elevations between approximately 0 m and +1 m relative to mean sea level, while it is rarely observed in areas below −0.4 m (Fig. S11). Similar results have been reported along the Jiangsu coast, where *S. alterniflora* distribution corresponds to areas with inundation probability lower than 0.4 (50).

These findings indicate that the seaward expansion of *S. alterniflora* is constrained by tidal inundation conditions and does not continue indefinitely across tidal flats. Modeling studies further suggest that under RCP4.5 and RCP8.5 climate scenarios, the potentially suitable habitat area for *S. alterniflora* along the Chinese mainland coast may increase by approximately 22.8% and 24.2% by the 2050s, respectively (51).

However, continued sea-level rise may limit expansion in lower intertidal zones, while artificial coastal structures restrict landward migration.

Taken together, these results indicate that the potential distribution space of *S. alterniflora* is environmentally constrained and may be manageable through spatially differentiated strategies.

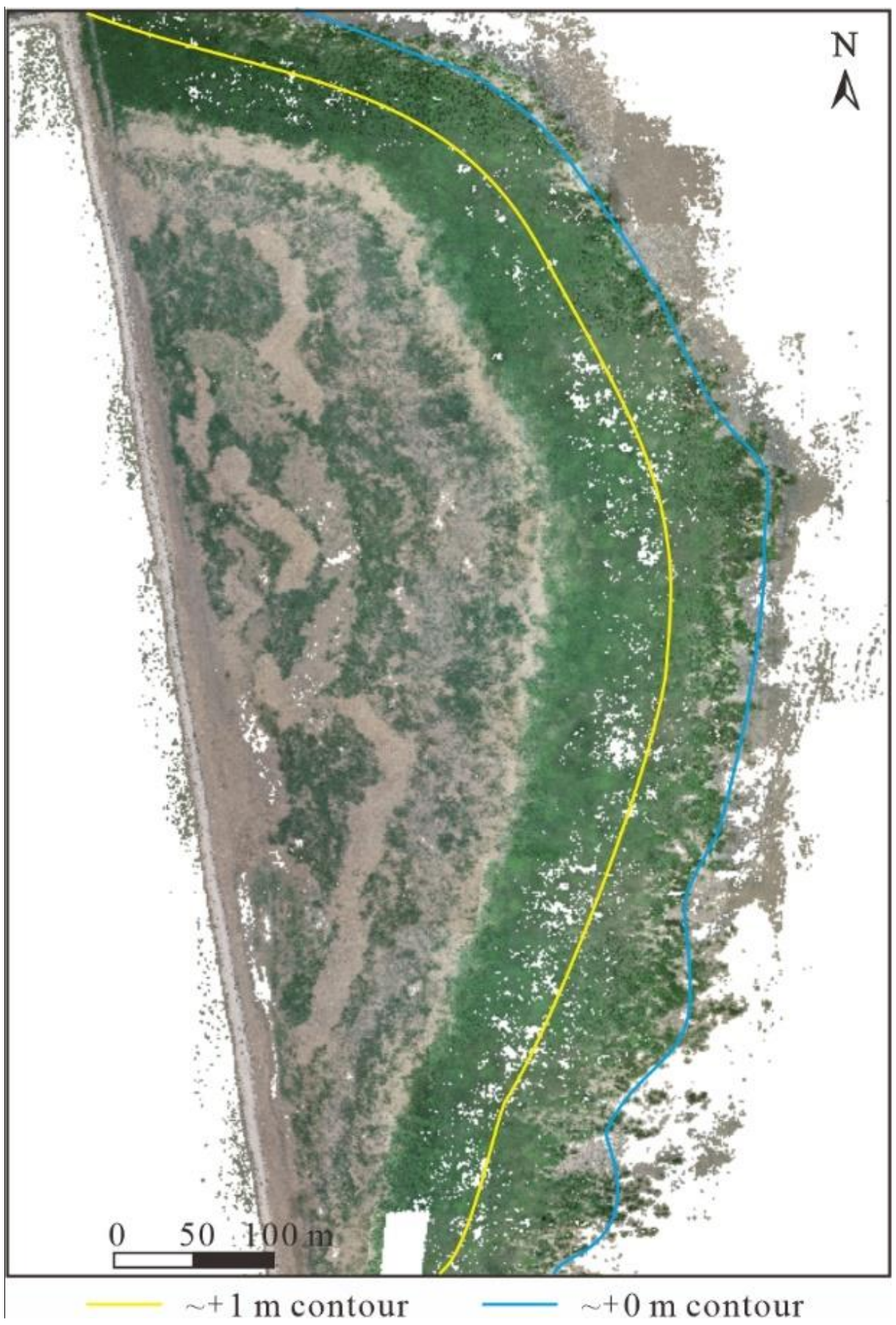

Fig. S12. Distribution and elevation range of *S. alterniflora* on the central Bohai Bay coast.

S6. Limitations and Uncertainties

This paper synthesizes published literature, policy documents, and our long-term field observations to evaluate the ecological trade-offs associated with *Spartina alterniflora* management across China's coastal regions. Several limitations should be acknowledged. First, the evidence base is spatially uneven, with relatively intensive field observations available for selected areas (e.g., Bohai Bay and Jiangsu coast), while comparable long-term datasets remain limited for many other regions along China's 18,000-km mainland coastline. As a result, the magnitude and consistency of ecosystem services such as sediment accretion, habitat provision, and carbon sequestration may vary across geomorphological and climatic gradients. Second, most existing studies, including our own field observations, are based on site-specific or short- to medium-term datasets, which may not fully capture long-term ecological dynamics, particularly under accelerating sea-level rise, climate warming, and changing sediment supply. Third, the relative contributions of *S. alterniflora* to coastal protection, biodiversity, and biogeochemical cycling remain context dependent, and uncertainties remain regarding the scalability of local observations to regional or national policy design. In addition, potential socioeconomic, governance, and implementation constraints influencing adaptive management effectiveness are not comprehensively assessed in this study. Therefore, our proposed adaptive strategy should be interpreted as a conceptual and evidence-informed framework rather than a universally prescriptive solution. Future research integrating standardized monitoring networks, long-term ecological observations, and coupled socio-ecological assessments will be essential to refine region-specific management thresholds and reduce uncertainty in balancing invasion risks with ecosystem service benefits.

Figure legends

1. photo source: https://scs.mnr.gov.cn/scsb/hykp/ 202006/ 5961819 d42be4cb98546e5 dce7faa439.shtml.
2. right photo source: http://www.guanhai.com.cn/p/115476.html.
3. photo source: https://www.cal-ipc.org/project/invasive-spartina-eradication/.
4. photo source: https://mp.weixin.qq.com/s?biz=MjM5MzgwNzM0MA==&mid=2651677839&idx=1&sn=18210d522b600b8d872c19ff8ca84031&chksm=bd68aa0c8a1f231a0134f35da565992bbf15959dd6a5c6ba431a8550326cae4f8735c3760e21&scene=27.

References

1. Chevalier, A. *Bulletin de la Société Botanique de France*, 70, 54–63 (1923).
2. Bortolus, A. et al. *Diversity and Distributions*, 21(11), 1267–1283 (2015).
3. Strong, D. R. et al. *University of California Press*, Berkeley and Los Angeles, 3–22 (2009).
4. Boston, K. The introduction of *Spartina townsendii* (S.L.) to Australia. Melbourne State College, Australia (1981).
5. Partridge, T. R. *New Zealand Journal of Botany*, 25, 4, 567–575 (1987).
6. Chung, C. H. *Ecological Engineering*, 2(3), 261–289 (1993).
7. Kim, S. et al. *Aquatic Invasions*, 18(3), 331–349 (2023).
8. Adams, J. B. et al. *South Africa African Journal of Marine Science*, 34(3), 313–322 (2012).
9. Sarkar, M. S. I. et al. *International Journal of Pollution and Noise Control*, 4(2), 6–18 (2018).
10. Tamaoki, M. et al. *Journal of Japan Society on Water Environment*, 38 (2), 61–66 (2015).
11. Crooks, J. A. *Oikos*, 97, 153–166 (2002).
12. Ministry of Agriculture and Rural Affairs of the People's Republic of China. *http://www.fgs.moa.gov.cn/flfg/202211/t20221109_6415160.htm* (2023).
13. Stokstad, E. *Science* 379, 6636 (2023)
14. Kerr, D. W. et al. *Biological Invasions*, 18(8), 2247–2266 (2016).
15. Cheng, B. B. et al. *Acta Agriculturae Shanghai*, 36(3), 90–95 (2020).
16. Janine, A. et al. *Biological Invasions*, 18(8), 2153–2158 (2016).
17. Xie, B. H. et al. *Chinese Journal of Applied Ecology*, 29(10), 3464–3476 (2018).
18. Zhao, Z. Y. et al. *Water Science and Engineering*, 13(1), 24–33 (2020a).
19. Li, S. H. et al. *Ecological Engineering*, 166, 106246 (2021).
20. Meng, K. H. et al. *Forestry Science and Technology*, 48(1), 20–24 (2023).
21. Wang, X. Y. *Public network*. *https://www.163.com/dy/article/HO10AS3A0514R9KU.html* (2022).
22. Mendelssohn, I. A. et al. *Concepts and controversies in tidal marsh ecology*, 2000, 59–80.
23. Knott, C. A. et al. *Journal of Aquatic Plant Management*, 51, 132–135 (2013).
24. Marriott, T. et al. B.C. *Spartina* treatment plan (June 2025–June 2028). Government of British Columbia, BC *Spartina* Working Group, Victoria, BC, Canada (2025).
25. Li, B. et al. *Ecological Engineering*, 35(4), 511–520 (2009).
26. Wan, S. et al. *Chinese geographical science*, 30, 202–216 (2020).
27. Wang, K. et al. *Journal of Cleaner Production*, 398, 136556 (2023).
28. Feng, J. et al. *Wetlands*, 34(4), 673–683 (2014).
29. China Green Express. *https://mp.weixin.qq.com/s/U8nyi-rra3o0h6WDuCUTzw* (2024).
30. Guo, H. P. et al. *Geology in China*, 44(6), 1115–1127 (2017).
31. IPCC, *Climate change 2021: the physical science basis*, 1–41 (2021).
32. MNR, Sea level bulletins 2022. Web site: http://www.nmdis.org.cn/hygb/zghpmgb/ (2023).
33. Wang, F. et al. *Geology in China*, 50(1), 61–83 (2023).
34. Wang, F. et al. *China Geology*, 1, 26–39 (2019).
35. Sun, Z. Q. et al. *North China Geology*, 47(1), 21–25 (2023).
36. Wang, F. et al. *China Geology*, 7, 1–11 (2024).
37. Wang, F. et al. *Science China: Earth Sciences* 61, 1510–1522 (2018).
38. Wang, F. et al. *J Coast Res* 294, 379–388 (2014).
39. Wang, A. J. et al. *Acta Oceanologica Sinica* 28, 92–99 (2006).
40. Wang, Y. P. et al. *Marine Geology* 291–294, 147–161 (2012).
41. Sha, W. X. et al. *Acta Ecologica Sinica*, 44(17), 1–10 (2024).
42. Lu, K. et al. *Ecosphere*, 13(3), e3963 (2021).
43. Xie, W. J. et al. *Ecological Engineering*, 35, 1158–1166 (2009).
44. Chen, Y. et al. *Ecological Applications*, *30*(3), e02056 (2020).
45. Li, B. et al. *Ecological Engineering, 35*(4), 511–520 (2009).
46. Gan, X., et al. *Marine Ecology Progress Series, 385*, 67–78 (2009).
47. Wang, Q., et al. *Biodiversity and Conservation, 25*(12), 2289–2305 (2016).
48. Ma, Z., et al. *Estuarine, Coastal and Shelf Science, 140*, 78–86 (2014).
49. Wang, F. et al. *News Letters of China Geological Survey*, 6 (16/17), 8–10 (2018).
50. Li, R. et al. *Estuarine, Coastal and Shelf Science*, 213, 305–313 (2018).
51. Chen, S. M. *Journal of Ecology and Rural Environment*, 37(12), 1575–1585 (2021).
